# PFEIFER: Preprocessing Framework for Electrograms Intermittently Fiducialized from Experimental Recordings

**DOI:** 10.21105/joss.00472

**Published:** 2018

**Authors:** Anton Rodenhauser, Wilson W Good, Brian Zenger, Jess Tate, Kedar Aras, Brett Burton, Rob S. MacLeod

**Affiliations:** 1 Scientific Computing and Imaging Institute, University of Utah, Salt Lake City, UT, USA; 2 Bioengineering Department, University of Utah, Salt Lake City, UT, USA

## Summary

### High Level Functionality

Preprocessing Framework for Electrograms Intermittently Fiducialized from Experimental Recordings (PFEIFER) is a MATLAB Graphical User Interface designed to process bioelectric signals acquired from experiments.

PFEIFER was specifically designed to process electrocardiographic recordings from electrodes placed on or around the heart or on the body surface. Specific steps included in PFEIFER allow the user to remove some forms of noise, correct for signal drift, and mark specific instants or intervals in time (fiducialize) within all of the time sampled channels. PFEIFER includes many unique features that allow the user to process electrical signals in a consistent and time efficient manner, with additional options for advanced user configurations and input. PFEIFER is structured as a consolidated framework that provides many standard processing pipelines but also has flexibility to allow the user to customize many of the steps. PFEIFER allows the user to import time aligned cardiac electrical signals, semi-automatically determine fiducial markings from those signals, and perform computational tasks that prepare the signals for subsequent display and analysis.

### Statement of Need

Time signals recorded from typical experiments in cardiac electrophysiology require a substantial amount of processing before they can be used for diagnostic or analytical purposes (Good et al. 2016, R. S. MacLeod et al. (2005), D. J. Swenson et al. (2011), Aras et al. (2014), Aras et al. (2016)). However, these processing steps are rarely described in adequate detail in literature. Until now, there has also been a scarcity of commonly available software tools, each group creating one-off tools for their exclusive use. This lack of shared tools, code, and even detailed algorithms has made comparisons across labs impossible. Rather then refining and improving these techniques, each lab has been required to traverse their own learning curve, repeat mistakes of others, and produce results that lack the confidence of robust and well tested processing.

PFEIFER provides both a set of open source tools to share with other groups as well as a common framework within which these processing steps can be performed. Because the framework is modular, flexible, and open source, written in a simple, high level language, other groups can easily replace or add functionality to suit there needs and compare results with other groups using the same tools. In addition to flexibility, PFEIFER has very little computational overhead so that it can efficiently process tens and even hundred of independent channels of time signals.

Of special note is that PFEIFER also contains built in functionality for significantly reducing overall experiment processing time by semi-automatically fiducializing the time signals, which are assumed to be approximately periodic, e.g., a sequence of heart beats. This capability is novel and we know of no other published description that achieves these goals. The combination of flexibility, minimal computational requirements, time saving algorithms, and a structured framework easy to modify and extend makes PFEIFER the ideal toolkit for researchers processing cardiac electrical signals.
